# Impact of Polymerase Fidelity on Background Error Rates in Next-Generation Sequencing with Unique Molecular Identifiers/Barcodes

**DOI:** 10.1038/s41598-019-39762-6

**Published:** 2019-03-05

**Authors:** Stefan Filges, Emiko Yamada, Anders Ståhlberg, Tony E. Godfrey

**Affiliations:** 1 Department of Surgery, Boston University School of Medicine, 700 Albany Street, Boston, MA 02118 USA; 2 Department of Pathology and Genetics, Sahlgrenska Cancer Center, Institute of Biomedicine, Sahlgrenska Academy at University of Gothenburg, Medicinaregatan 1F, 405 30 Gothenberg, Sweden; 3 Wallenberg Centre for Molecular and Translational Medicine, University of Gothenburg, Gothenburg, Sweden; 4 Department of Clinical Pathology and Genetics, Sahlgrenska University Hospital, 413 45 Gothenburg, Sweden

## Abstract

Liquid biopsy and detection of tumor-associated mutations in cell-free circulating DNA often requires the ability to identify single nucleotide variants at allele frequencies below 0.1%. Standard sequencing protocols cannot achieve this level of sensitivity due to background noise from DNA damage and polymerase induced errors. Addition of unique molecular identifiers allows identification and removal of errors responsible for this background noise. Theoretically, high fidelity enzymes will also reduce error rates in barcoded NGS but this has not been thoroughly explored. We evaluated the impact of polymerase fidelity on the magnitude of error reduction at different steps of barcoded NGS library construction. We find that barcoding itself displays largest impact on error reduction, even with low fidelity polymerases. Use of high fidelity polymerases in the barcoding step of library construction further suppresses error in barcoded NGS, and allows detection of variant alleles below 0.1% allele frequency. However, the improvement in error correction is modest and is not directly proportional to polymerase fidelity. Depending on the specific application, other polymerase characteristics such as multiplexing capacity, PCR efficiency, buffer requirements and ability to amplify targets with high GC content may outweigh the relatively small additional decrease in error afforded by ultra-high fidelity polymerases.

## Introduction

The introduction of next-generation sequencing has led to revolutionary capabilities in research and in clinical testing^1^. Sequencing of whole genomes, exomes or targeted genomic regions is now routine and variant and/or mutant alleles can be identified with much higher sensitivity than with Sanger sequencing. NGS sensitivity depends on multiple factors but detection of variants, in particular single nucleotide variants, that occur at a frequency below ~1% remains challenging due to background noise^2^. Errors in NGS result from multiple factors including DNA damage, polymerase induced errors during library construction and sequencer read errors^2–4^. The majority of these errors can be identified and removed by addition of unique molecular identifiers (UMI’s, often referred to as barcodes)^5–11^. Molecular barcoding of individual DNA template strands during NGS library construction results in the ability to track all sequencing reads back to a single original DNA template. By aligning reads with the same barcode, it is then possible to differentiate between true variants and those resulting from sequencer or *Taq* polymerase errors in any except the very first PCR cycle^5^.

There are now many published protocols for incorporation of barcodes into NGS libraries^12^, all of which require PCR amplification steps unless large amounts of DNA are available. Barcodes may be ligated onto target DNA followed by selection and amplification^5,9^ or they can be incorporated directly in the first 2–3 cycles of PCR followed by a second round adapter PCR to increase yield and incorporate sequences required for different sequencing instruments^5,11^. Most of these approaches report reduction of sequencing errors, and ability to detect true variants below 0.1% variant allele frequency (VAF).

**Figure 1:**
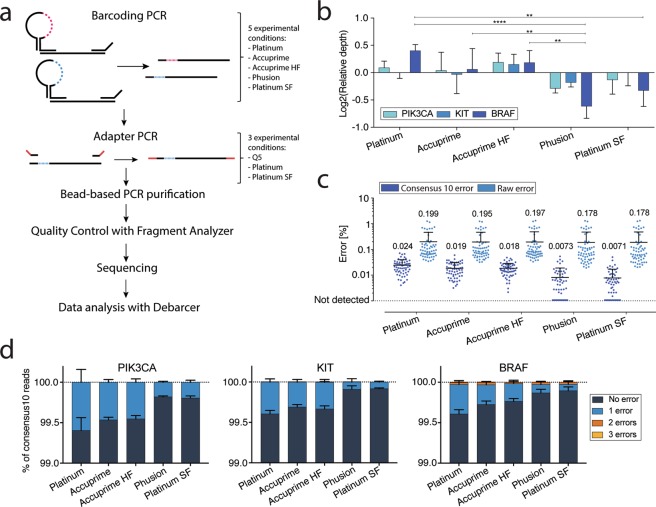
Effect of polymerase fidelity in the initial barcoding PCR. (**a**) Workflow for SimSen-Seq indicating which polymerases were tested in the barcoding and adapter PCR steps. Dotted lines represent barcodes with colors indicating different barcode sequences. Red lines indicate the P5 and P7 adapter sequences. (**b**) Relative consensus_10_ sequencing depths for each amplicon using five different polymerases in the barcoding PCR step (Q5 polymerase was used for all adapter PCRs). Consensus depths were compared using 2-way ANOVA (*p < 0.05; **p < 0.01; ***p < 0.001; ****p < 0.0001). (**c**) Comparison of raw and consensus_10_ errors observed at all base positions (all three amplicons combined) using five different polymerases in the barcoding PCR and Q5 polymerase in all adapter PCRs. No significant differences were found between raw errors. For the consensus_10_ error, all polymerases showed significantly lower error than Platinum Taq (Wilcoxon signed-rank test, p < 0.001). In addition, both Phusion and Platinum SuperFi polymerases had lower error rates than both Accuprime polymerases but were not significantly different from each other (p > 0.05). Each dot denotes a nucleotide in one of the 3 amplicons, Mean ± SD is shown (n = 3). (**d**) Total percentage of consensus_10_ reads with 0, 1, 2 or 3 variant bases. HF, High Fidelity; SF, SuperFi.

DNA polymerases have different properties affecting their PCR performance including specificity, thermostability, processivity and fidelity^13–15^. Furthermore, replication fidelity depends on factors such as nucleotide selectivity and proofreading capability^16–18^. Fidelity can be defined in several ways but for simplicity, it is often reported as fidelity relative to that of *Taq* polymerase. Most NGS library construction protocols use high fidelity polymerases to minimize errors and several studies have evaluated this in standard NGS protocols. Theoretically, high fidelity enzymes will also reduce error rates in barcoded NGS but the magnitude of this reduction and the importance of high fidelity enzymes at different steps of library construction have not to our knowledge been reported. In this study, we evaluate the impact of polymerase fidelity in a PCR-based barcoding approach published previously^11^. SimSenSeq (SSS) consists of an initial 3-cycle PCR step, during which unique barcodes are incorporated into the PCR products, followed by an adapter PCR step which adds the Illumina P5 and P7 primer sequences to the library (Fig. 1a)^19^. We tested five different polymerases with fidelity ranging from 1X to >100X in both the barcoding and adapter PCR steps and evaluated the impact on both raw sequencing error and error corrected reads using the barcodes (consensus error). In addition, we compared error types and sequence context dependence of errors with the different polymerases. Our results provide practical insights into the value of using high fidelity polymerases at different steps during barcoded NGS library construction.

## Results

### Effect of polymerase fidelity in the initial barcoding PCR

SimSenSeq consists of an initial 3-cycle PCR step, during which unique barcodes are incorporated into the PCR products, followed by an adapter PCR step which adds the Illumina P5 and P7 primer sequences to the library (Fig. 1a). First, we explored the effect of five different polymerases on library read depth (Fig. 1b) and on both raw and barcode corrected (consensus_10_) sequencing error rates when used in the barcoding PCR step (Fig. 1c). While the highest fidelity polymerases tended to have slightly lower read depths for all amplicons, this was only statistically significant for the BRAF amplicon and for specific polymerase comparisons as indicated (Fig. 1a). The largest difference noted was for Platinum vs Phusion polymerase where the BRAF amplicon relative read depth was 2.1-fold lower for Phusion.

When comparing raw read error rates, we found no significant difference between any of the polymerases. However, when using barcode correction (consensus_10_), error rates gradually dropped as polymerase fidelity increased, resulting in a 3.9-fold error reduction for Platinum vs Platinum SuperFi. All polymerases resulted in significantly lower error rates than the standard fidelity Platinum Taq and the two highest fidelity polymerases, Phusion and Platinum SuperFi both resulted in significantly less error than all three lower fidelity polymerases, but were not different from each other. The observed effect of different polymerase fidelity in the barcoding PCR step of SSS indicates that raw error is predominantly the result of errors introduced in the adapter PCR or on the sequencer during cluster formation and/or sequence reading. These errors are corrected using the consensus_10_ data, allowing the effect of polymerase fidelity to be observed.

Next, we looked at the effect of polymerase fidelity on the percentage of consensus_10_ reads that had zero, one, two or three errors in each amplicon (Fig. 1d). For all amplicons, it is clear that the number of reads with zero errors increases with increasing polymerase fidelity while the number of reads with one error decreases. Reads with two errors are extremely rare in the PIK3CA and KIT amplicons but were consistently noted at ~0.025% of the total reads for the BRAF amplicon, regardless of the polymerase used. Further exploration of the BRAF data identified that the majority (all but 2) of the two error reads were due to CA to TT or AC to TT tandem errors at the first and second or second and third bases of BRAF codon 600 (Supplementary Table 1). When examining other data for the BRAF amplicon, generated using cell-free plasma DNA, cell line DNA and normal tissue DNA tested in ongoing projects, we found similar frequencies of these same tandem mutations in all samples (data not shown). Finally, we explored the COSMIC database for BRAF and again identified both TT tandem mutations reported (Supplementary Table 1), indicating that our data is not a technical artifact of SSS.

### Higher Fidelity Polymerases Correct A to G or T to C Transitions

**Figure 2:**
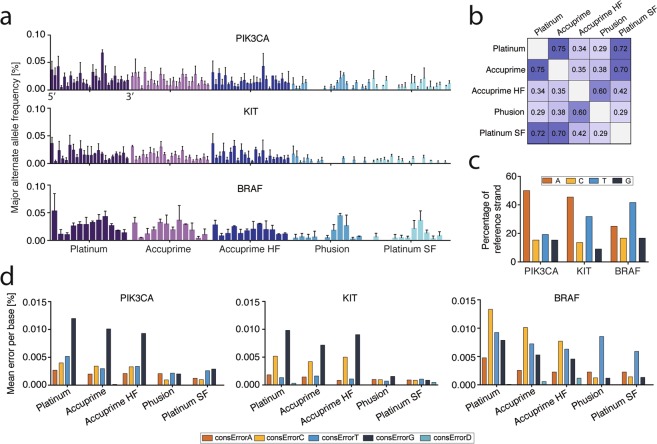
Sequence dependent errors. (**a**) Comparison of consensus_10_ error at each base using different polymerases in the barcoding PCR step. Mean ± SD is shown (n = 3). (**b**) Spearman’s correlation coefficient between error frequencies by base position and different polymerases using different polymerases in barcoding PCR. All correlations are significant (p < 0.05). (**c**) Base composition of the reference strand in each amplicon. (**d**) Mean consensus_10_ error in each amplicon by nucleotide change relative to the reference sequence. For example, consErrorG of 0.01% reflects that 0.01% of bases were changed to a G from any of the other bases. The observed pattern is congruent with expected polymerase-induced errors based on the base composition of each amplicon. consErrorD denotes deleted bases. HF, High Fidelity; SF, SuperFi.

Figure 2a shows consensus 10 error rates observed at each base position in each amplicon, and the effect of using different polymerases during the barcoding PCR (Absolute counts in Supplementary Fig. 1). Similar patterns of error frequency are observed but with different magnitudes of error. Indeed, we found significant correlation between error rate and base position for all polymerase combinations, indicating some degree of sequence context dependence that is similar across polymerases (Fig. 2b and Supplementary Fig. 2). Exploring this data further, we determined the base composition of each amplicon (Fig. 2c) and the types of errors introduced for each amplicon and polymerase. The most common errors observed were A to G transitions. A to G transitions predominated for both the PIK3CA and KIT amplicons, both of which contain a high A percentage in the reference strand. Correction of these transition errors by Phusion and Platinum polymerases is responsible for the significantly lower error rates observed for these two enzymes (Fig. 2d and Supplementary Fig. 3). The same is also true for the BRAF amplicons, although T to C transitions are more common (and are corrected by Phusion and Platinum SuperFi) since this amplicon is more T-rich. However, the BRAF amplicon also shows a relatively high A, C or G to T (consT) error that is not corrected by any polymerase. This high, uncorrected error is due to the tandem TT base changes discussed above and the fact that it cannot be corrected indicates either a non-polymerase related source of error such as physical base modification during processing or the real presence of these sequences at a very low level in the target DNA.

### Effect of low versus high-fidelity polymerase in the adapter PCR

**Figure 3:**
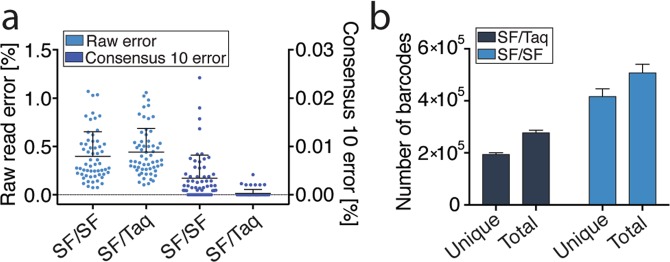
Effect of polymerase fidelity in the adapter PCR. (**a**) Comparison of raw and consensus_10_ errors for Platinum SuperFi (SF) and regular Taq polymerase (Platinum) in the adapter PCR. (**b**) Number of observed barcodes depends on the polymerase used in the adapter PCR.

Next we evaluated the effect of using regular Platinum Taq (1X fidelity) versus Platinum SuperFi polymerase (>100X fidelity) in the adapter PCR. In both cases, Platinum SuperFi was used for the barcoding PCR step. Figure 3a shows the raw and consensus_10_ error for both polymerase combinations. No significant difference was found for either raw or consensus_10_ error, once again indicating that the majority or errors do not occur in the NGS library preparation but in subsequent procedures. Interestingly, regular Platinum Taq appears to give a slightly lower consensus_10_ error rate than Platinum SuperFi but this is most likely an artifact as Platinum Taq polymerase amplified approximately 50% fewer unique barcodes than Platinum SuperFi (Fig. 3b and Supplementary Fig. 4); resulting in artificially reduced sensitivity for the consensus_10_ analysis.

### Mutant detection sensitivity with high-fidelity polymerases

**Figure 4:**
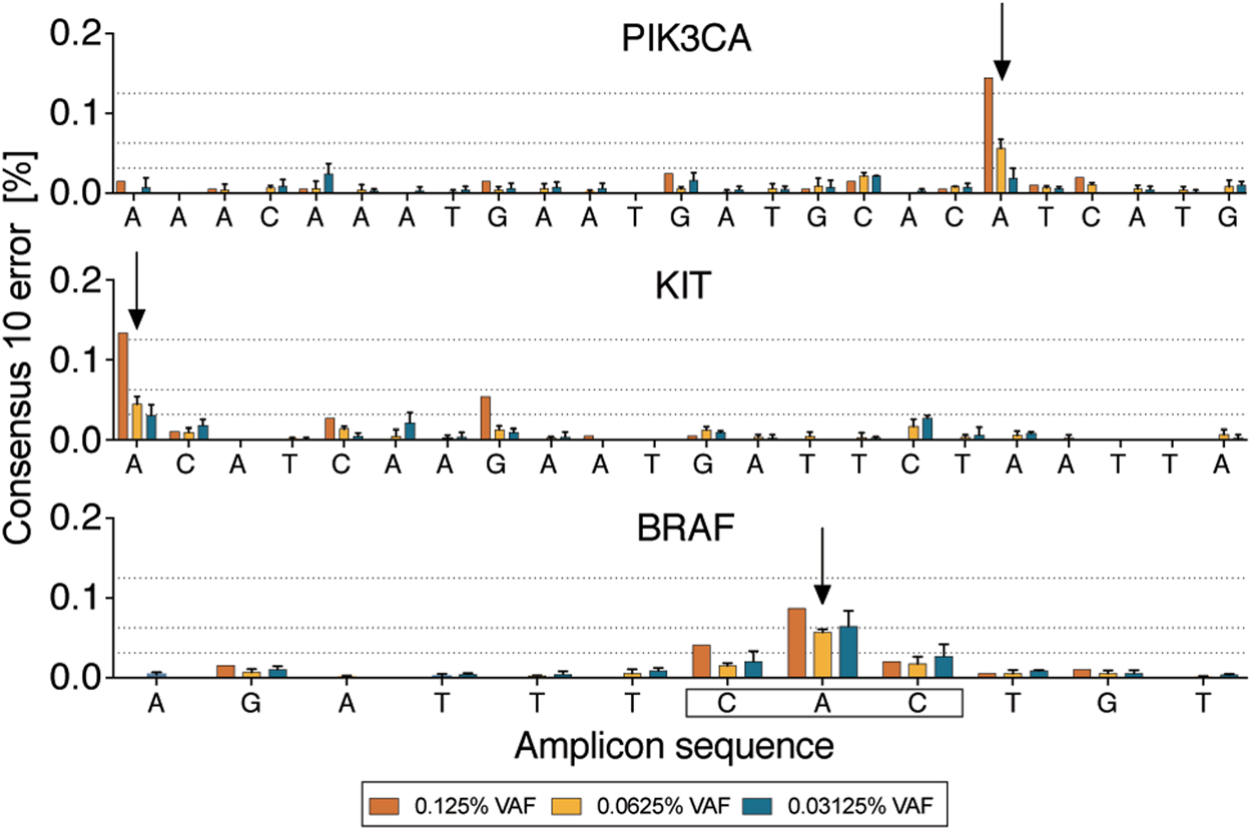
Determination of SiMSen-Seq sensitivity. Seraseq ctDNA Mutation Mix v2 reference DNA containing known variant alleles in PIK3CA, KIT and BRAF at allele frequencies of 0.125%, 0.0625% and 0.03125% were sequenced with SiMSen-Seq. Arrows indicate the position of the expected variants. The reading frame in the BRAF amplicon indicates codon V600. Consensus_10_ errors are all non-reference bases present in ≥90% of reads in barcode families with at least 10 reads.

Finally, we assessed the sensitivity of SimSenSeq for mutant detection when using the highest fidelity polymerase (Platinum SuperFi) in both rounds of PCR. Detection of BRAF V600E, KIT D816V and PIK3CA H1047R was tested at 0.125% (n = 1), 0.0625% (n = 3) and 0.0313% (n = 3) VAF. As shown in Fig. 4, for KIT and PIK3CA, presence of mutations at 0.125% (~19 mutant copies) and 0.0625 (~15 mutant copies) are clearly seen above background error rates while 0.0313 (~8 mutant copies) is not detected above background. Expected numbers of mutant copies are based on the amount of genome equivalents loaded into the barcoding reaction, assuming that 1 ng of genomic DNA contains 278 haploid genomes. Absolute counts are shown in Supplementary Fig. 5. Presence of the tandem TT mutations in the BRAF template DNA precludes analysis of sensitivity for this amplicon.

## Discussion

Here, we evaluate the importance of polymerase fidelity on correction of sequencing error when using barcodes. Our data demonstrate that barcoding effectively allows identification and removal of errors introduced after the barcoding step *i.e*., during library amplification or on the sequencer itself, regardless of the fidelity of the polymerase used for library amplification. Although higher polymerase fidelity resulted in slightly reduced raw sequencing error, this was not statistically significant, indicating that raw error is predominantly a result of errors generated on the sequencer rather than in the library preparation.

Our data show that even with lower-fidelity polymerases such as Platinum Taq (1X fidelity), Accuprime (2X) and Accuprime HiFi (9x) in the barcoding step, barcoding results in approximately 9–10 fold error reduction, compared to the raw error, while Phusion (50X and Platinum HiFi (>100X) result in approximately 25-fold lower error. On the other hand, higher polymerase fidelity during the barcoding steps had a significant effect on consensus error, although not as much as one might assume. While statistically significant, differences in consensus error rates between Accuprime or Accuprime HiFi and Platinum Taq were not practically meaningful (~1.3-fold reduction). In comparison, Phusion and Platinum HiFi polymerases both resulted in approximately 3.9-fold consensus error reduction compared with Platinum Taq. However, these correction factors are likely dependent on the specific method used for barcoding, the amount of DNA coverage of the assays used and on the number of PCR cycles performed in the barcoding step. For example, if more PCR cycles were used in the barcoding step then presumably one would see a larger effect of polymerase fidelity on the consensus error rates as there is more potential for errors to occur during the barcoding step. While the number of bases interrogated in this study is relatively small, our data is similar in scope to that which may be obtained from clinical gene panels where short amplicons target only a limited number of actionable mutations in highly fragmented cfDNA. Regardless, the data presented have important implications regarding enzyme choice for PCR-based barcoding strategies. Higher fidelity polymerases are typically more expensive than lower-fidelity, more generic polymerases and may be unnecessary for some applications. Furthermore, polymerases have other properties that may influence choice depending on the application^4,20^. For example, some polymerases are better for multiplexing, PCR efficiencies and robustness of amplification can be different, buffer requirements are different and may be incompatible with some applications, some work better with high GC content amplicons etc. In some cases these considerations may outweigh the relatively small additional decrease in error afforded by ultra-high fidelity polymerases.

Along these lines, our data comparing different polymerases for library amplification (adapter PCR) are also informative. Here, we found that higher polymerase fidelity in the adapter PCR does not result in reduced consensus error. In fact, consensus error was unexpectedly lower with Platinum Taq than with Platinum HiFi in the adapter PCR and the explanation for this underscores how polymerase properties other than fidelity can impact quality of the data. We found that the number of unique barcodes (represented by at least 10 reads) in the libraries generated with Platinum Taq was approximately half that generated using Platinum HiFi. This indicates amplification bias and means that many of the original DNA molecules that are barcoded in the initial PCR are not being amplified in the adapter PCR. This is problematic as rare mutant DNA fragments may not be represented in the final library, resulting in reduced sensitivity, while also artificially suppressing observed error rates. Thus, for the adapter PCR where high-level amplification is required, it may be better to choose a polymerase with low amplification bias and high efficiency rather than high fidelity. In our experiments the higher fidelity polymerase also happened to have less bias but other polymerases may be even better and/or less expensive.

In summary, our data indicates that while high polymerase fidelity reduces error in barcoded NGS, improvement in error correction is not directly proportional to fidelity and is not the only factor that should be considered. For some applications, polymerases with lower fidelity may provide adequate error correction while providing other beneficial characteristics.

## Materials and Methods

### Polymerases

To evaluate the effect of polymerase fidelity on error rates in a barcoded NGS library construction protocol (SSS) we selected five polymerases with fidelities ranging from 1X to >100X that of standard Taq polymerase. Fidelity estimates were taken directly from product literature available from the supplier. The following polymerases were selected, all from Thermo Fisher Scientific; Platinum Taq (1X fidelity), AccuPrime Taq (2X fidelity), Accuprime Taq DNA polymerase, high fidelity (9X fidelity), Phusion II Hot Start Taq (52X Fidelity) and Platinum SuperFi polymerase (>100X fidelity).

### DNA sources

DNA was obtained from SeraCare, Milford MA. Two DNA sources were used, one “wild-type” control DNA (Seraseq ctDNA Mutation Mix v2 WT cat# 0710-0144) and one mutant mix starting at 0.125% VAF (Seraseq ctDNA Mutation Mix v2 AF 0.125% cat# 0710-0143). Both were derived from DNA from the GM24385 cell line (Coriell). Variants in the 0.125% VAF sample were added using synthetic sequences, and, as part of manufacture, the WT and 0.125% VAF mixtures were subjected to additional processing including fragmentation by sonication and size selection. The wild-type DNA was used for initial polymerase testing to determine impact of polymerase fidelity on background error rates while the mutant mix was used in sensitivity tests.

### SiMSenSeq Assays and NGS Library Preparation

Three well-performing SSS assays covering 59 bases were selected to match three mutations included in the SeraCare mutation panel. Primer sequences for these assays are shown in Supplementary Table 2 along with the specific mutations that are targeted in the Seraseq DNA. SSS was performed as described previously^10^ except that different polymerases were substituted in either the barcoding or adapter PCR steps as indicated in Fig. 1a. SSS libraries were generated for all three assays in the same reaction (triplex) and all polymerases were used at the recommended concentrations and in the supplied buffers (Buffer HF was used for Phusion polymerase). In addition, 0.5 M L-Carnitine was added to Phusion reactions and 0.05 mg/mL BSA was added for the Accuprime HiFi reactions as described previously^10^. 80 ng of wild-type Seraseq DNA was used in each assay (n = 3 replicates in all cases) except for the mutation detection sensitivity tests. For the sensitivity tests, 50 ng of the 0.125% VAF mutation DNA mix (n = 1) was used as a positive control. For the 0.0625% and 0.0313% VAF tests, the 0.125% mutation mix DNA was diluted in wild type DNA and 80 ng of DNA was used in each assay and each assay was run in triplicate.

Adapter PCR products were purified using the Agencourt AMPure XP system (Beckman Coulter, Inc) according to the manufacturers’ instructions. The applied volume ratio between beads and PCR products was 1:1 and the purified product was eluted in 20 μL of 10 mM Tris buffer, pH 8.0. Prior to sequencing, library products were assessed on a Fragment Analyzer (Advanced Analytical) to ensure correct sizing.

### Library Sequencing

The products from the second round of PCR contain Illumina sequencing adaptor sequences and indexes and were sequenced on either MiSeq or MiniSeq instruments (Illumina) in single end 150 bp mode.

### Data analysis

Raw FASTQ files were aligned to hg19 using BWA-MEM (0.7.12) with output bam files sorted by position and indexed using SAMtools (0.1.19). Aligned reads were analyzed as described previously^10,11^ using a modified version of Debarcer (https://github.com/oicr-gsi/debarcer/releases/tag/v0.3.1) on a CentOS 6.9 cluster. Briefly, valid reads within each amplicon were identified as those which contained a barcode sequence in the correct orientation relative to the sequence of the targeting primer and hairpin stem. Reads were then grouped into families by amplicon and random 12mer barcode. For reads within each family, alignment information for individual reads was used to determine a consensus identity for bases (including indels) at each nucleotide position within the amplicons. This procedure is conceptually similar to that described in Schmitt *et al*.^6^. Non-reference sequences were reported in consensus sequences if they composed 100% of the reads in families with 10–20 reads, or at least 90% of reads in families with >20 reads. The Debarcer output files were further processed using custom R-scripts (R version 3.4.1) to calculate error frequencies for per base raw and consensus read counts; and to calculate fractions of non-reference bases in consensus reads.

### Statistical analyses

All statistical analyses were performed in Prism 7 for Mac OS X (Version 7.0a). Comparisons of consensus errors for the different enzymes were made using Wilcoxon signed-rank tests. Effectiveness of sample pairing was determined with Spearman correlation and was statistically significant for all comparisons made. Relative sequencing depths were compared using 2-way ANOVA with Tukey’s post-hoc analysis. All tests were considered significant at the α = 0.05 level. A summary of test results and P-values can be found in Supplementary Tables 3–5.

## Supplementary information

Supplementary Figures

Supplementary Table 1

Supplementary Table 2

Supplementary Table 3

Supplementary Table 4

Supplementary Table 5

## Data Availability

Raw sequencing data in FASTQ format is publically available from the NCBI Sequence Read Archive (SRA) under submission ID PRJNA50736.
