# Supplementary material for: Impact of Polymerase Fidelity on Background Error Rates in Next-Generation Sequencing with Unique Molecular Identifiers/Barcodes
Source: Sci Rep. 2019 Mar 5;9:3503. doi: 10.1038/s41598-019-39762-6 (PMC6401092; doi:10.1038/s41598-019-39762-6)
Supplement: Supplementary file 1 — Supplementary Figures [file 41598_2019_39762_MOESM1_ESM.pdf]

Supplementary Figures

Impact of Polymerase Fidelity on Background Error Rates in Next-Generation Sequencing with Unique Molecular Identifiers/Barcodes

Stefan Filges[2], Emiko Yamada[1], Anders Ståhlberg[2,3,4*], and Tony E. Godfrey[1*]

[1] Department of Surgery, Boston University School of Medicine, 700 Albany Street, Boston, MA 02118, USA

[2] Department of Pathology and Genetics, Sahlgrenska Cancer Center, Institute of Biomedicine, Sahlgrenska Academy at University of Gothenburg, Medicinaregatan 1F, 405 30 Gothenberg, Sweden

[3] Wallenberg Centre for Molecular and Translational Medicine, University of Gothenburg, Sweden.

[4] Department of Clinical Pathology and Genetics, Sahlgrenska University Hospital, 413 45 Gothenburg, Sweden.

* To whom correspondence should be addressed:

A.S. Tel: +46 31 7866735; email: anders.stahlberg@gu.se

T.E.G. Tel: +1 617 358 8494; email: godfreyt@bu.edu

Supplementary Figures

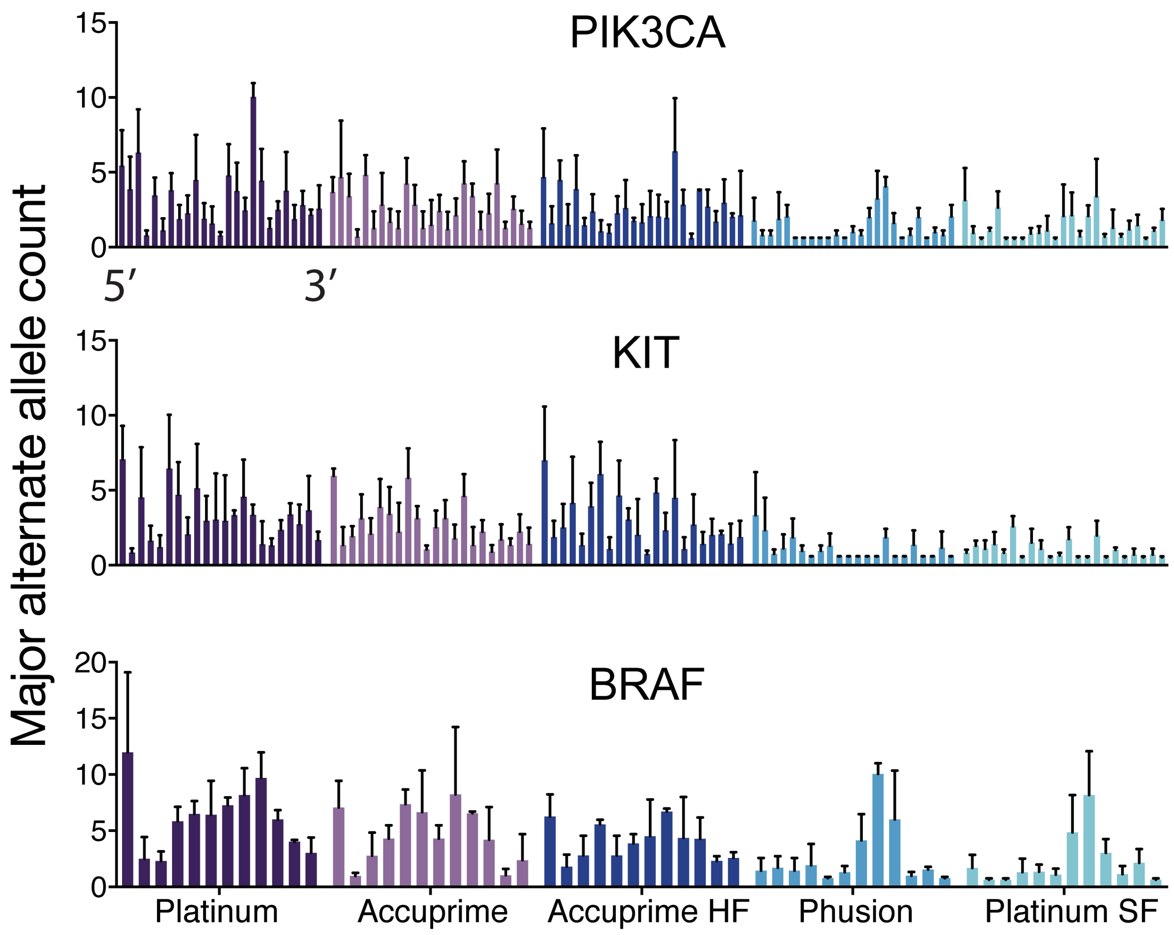

Supplementary Figure 1. Sequence dependent errors. Major alternate allele counts for consensus 10 error at each base using different polymerases in the barcoding PCR step. Mean ± SD is shown (n = 3). HF, High Fidelity; SF, SuperFi.

Supplementary Figures

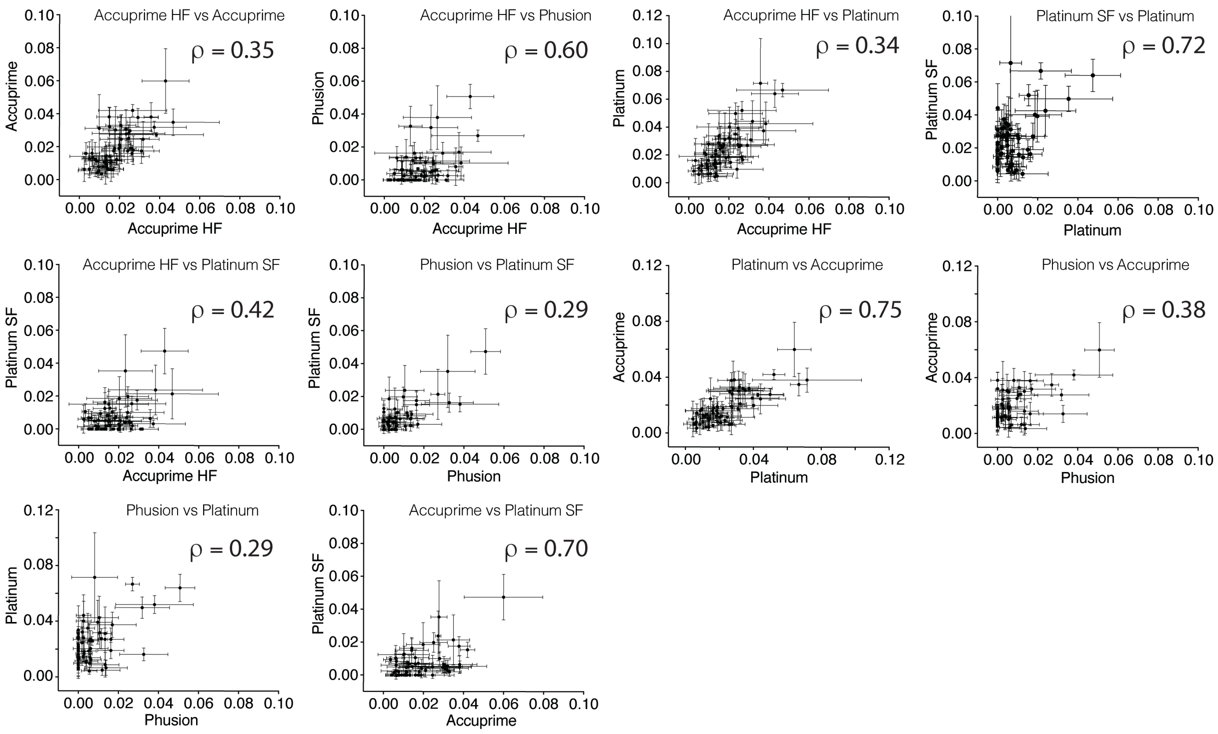

Supplementary Figure 2. Scatterplot of error frequencies by base position with different polymerases. Spearman's correlation coefficient (ρ) is shown for each comparison. All correlations were statistically significant ($p < 0.05$). Each dot represents a nucleotide position in one of the three amplicons. Mean ± SD is shown (n = 3). HF, High Fidelity; SF, SuperFi.

Supplementary Figures

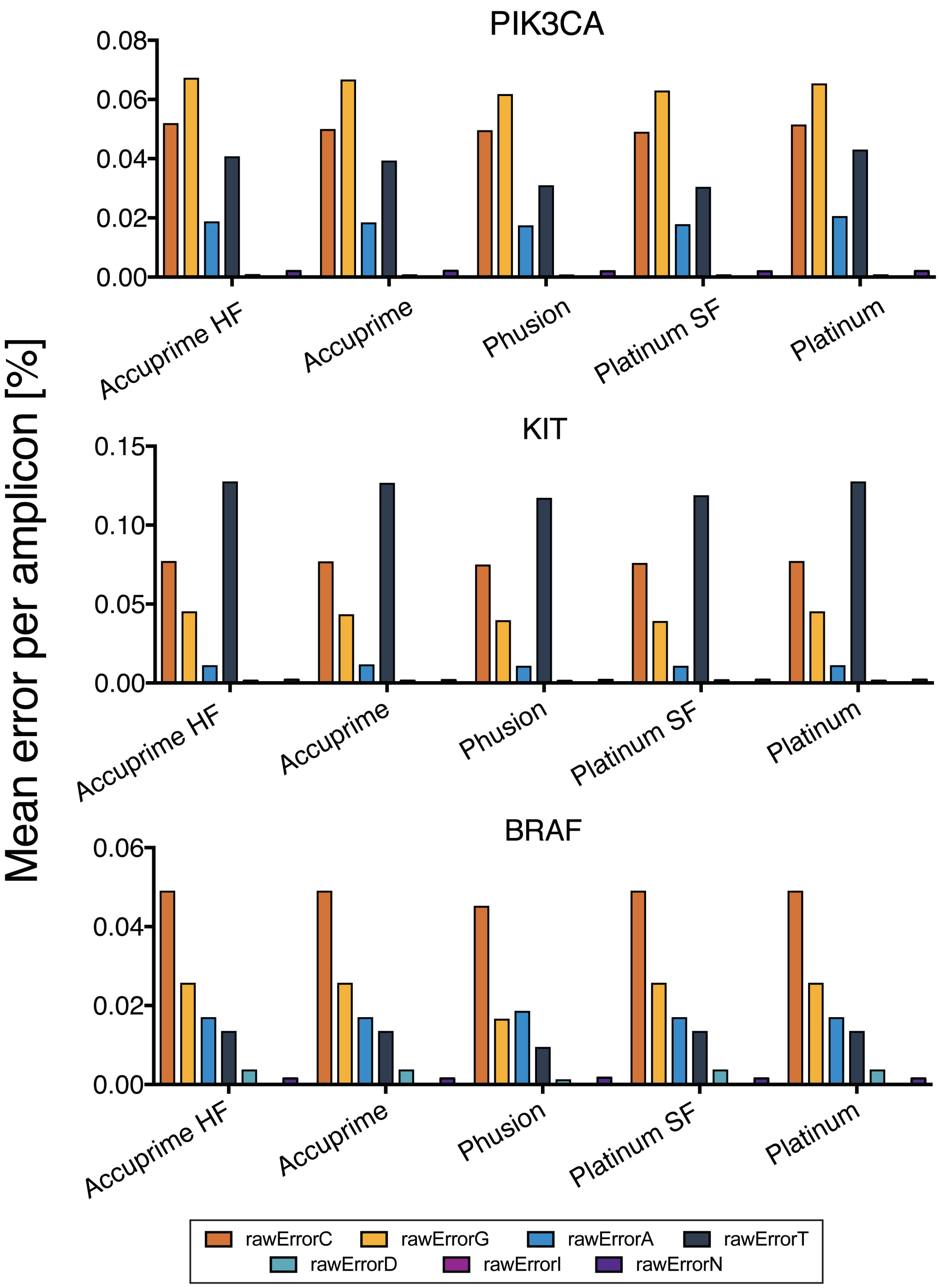

Supplementary Figure 3. Mean raw error in each amplicon by nucleotide change. For example, rawErrorG of 0.1% reflects that 0.1% of bases were changed to a G from any of the other bases. The observed pattern is congruent with expected polymerase-induced errors based on the base composition of each amplicon. rawErrorD, deletions; rawErrorI, insertions; rawErrorN, any base. HF, High Fidelity; SF, SuperFi.

Supplementary Figures

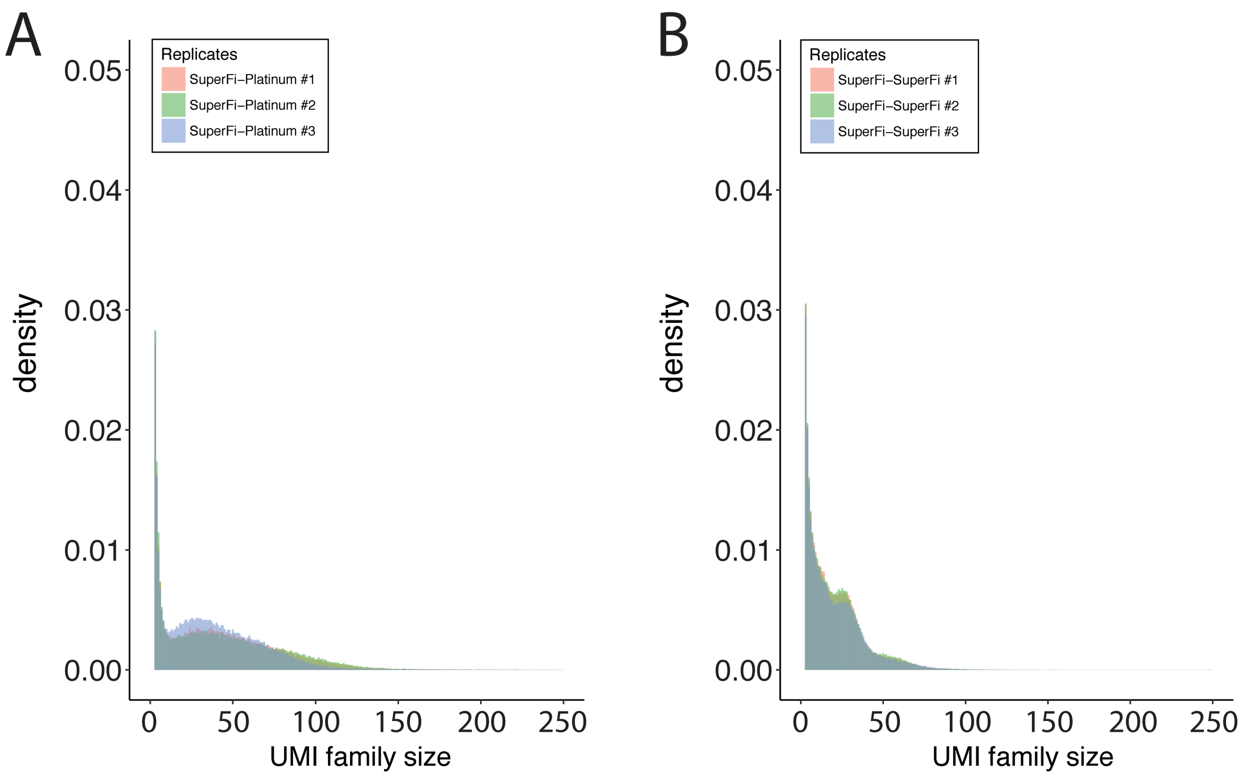

Supplementary Figure 4. Barcode density distributions. A) Low and **B)** high fidelity polymerases when used in the adapter PCR step.

Supplementary Figures

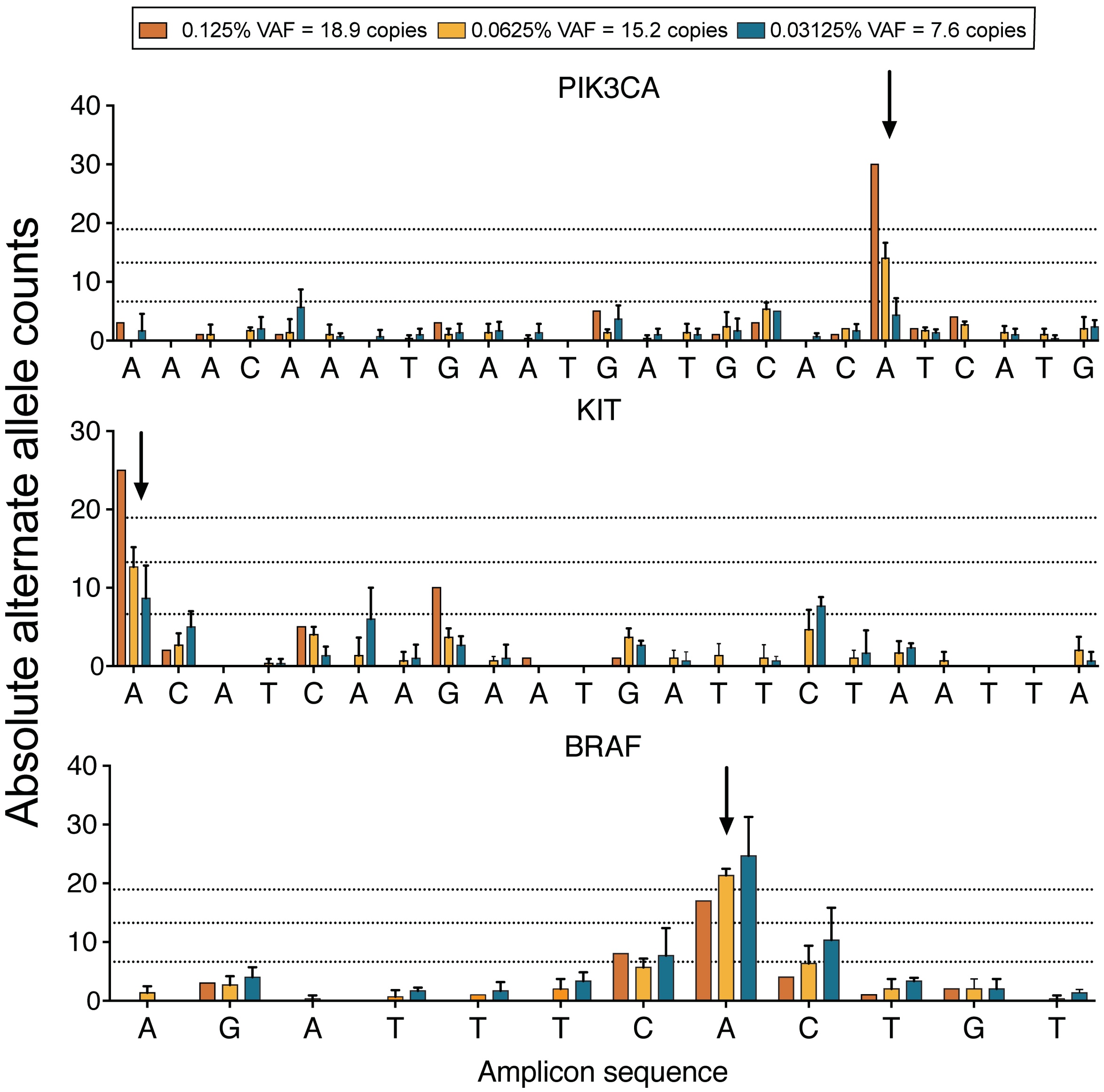

Supplementary Figure 5. Determination of SiMSen-Seq sensitivity. Known cell-free DNA-like standards containing variant alleles in PIK3CA, KIT and BRAF at allele frequencies of 0.125%, 0.0625% and 0.03125% were sequenced with SiMSen-Seq. Arrows indicate the position of the expected variants. The reading frame in the BRAF amplicon indicates codon V600. Compared to Figure 4, the absolute alternative allele counts are shown.
